# Aortic Bifurcation Saddle Thrombus

**DOI:** 10.7759/cureus.4752

**Published:** 2019-05-25

**Authors:** Casey Arnold, Carmen J Martinez Martinez

**Affiliations:** 1 Emergency Medicine, Advent Health Florida Hospital, Orlando, USA

**Keywords:** aortic thrombus, saddle thrombus, cardiac thrombus, intraventricular thrombus, acute aortic occlusion, acute aortoiliac occlusion

## Abstract

Acute aortic pathology demands a high index of suspicion and frequent reevaluations during emergency department (ED) stay for proper diagnosis. This high index of suspicion is crucial to avoid missing the potentially devastating aortic diagnosis. Here, we present a 59-year-old male who presented with chest pain and was ultimately diagnosed with a rare aortic bifurcation saddle thrombus causing acute aortic occlusion. This diagnosis, although rare, highlights a more common point that all patients should be reevaluated for an acute aorta, especially when diagnostic clues are present. The diagnosis was found only because of a thorough reevaluation. Missing the diagnosis would have resulted in death or lifetime dependence on hemodialysis.

## Introduction

An acute aortic pathology will bedevil even the most careful emergency physician. With a low overall incidence rate [1-2] and common alternative diagnoses to explain symptoms, the diagnosis of an acute aortic pathology is often overlooked. Aortic etiologies are often considered in our differential of acute chest or abdominal pain, but they are overshadowed by common alternative diagnoses because the rare aortic diagnoses always require the cumbersome contrast-enhanced computed tomography (CT) scan. Unfortunately, the consequences of missing the acute aorta are often grave, with aortic occlusions carrying a mortality rate of 21% even when they are diagnosed and treated promptly [3]. We present the case of a 59-year-old male with an acute aortic bifurcation saddle thrombus causing acute aortic occlusion, where a thorough reevaluation clinched the diagnosis in the ED.

## Case presentation

**Figure 1:**
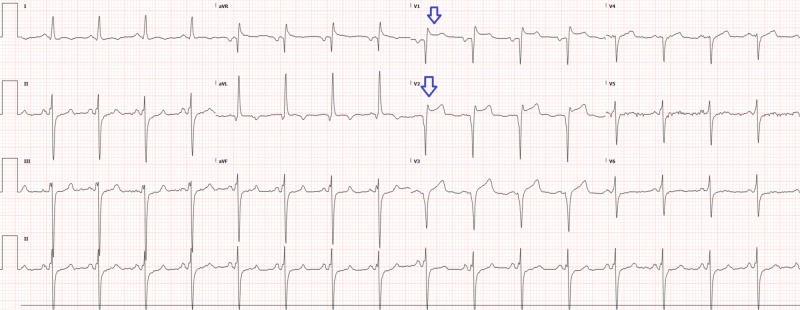
Electrocardiogram at triage showing ST elevations (arrows)

**Figure 2:**
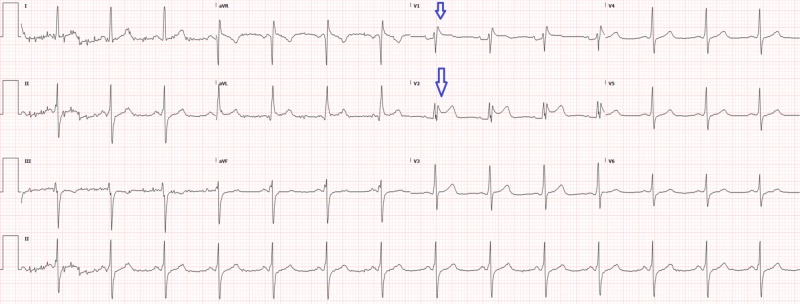
Old electrocardiogram in EMR from one year ago showing similar elevations (arrows) EMR: electronic medical record

A 59-year-old male with a past medical history of diabetes, hypertension, stroke, and congestive heart failure presented to the emergency department for epigastric pain, chest pain, and hyperglycemia. Initial vital signs were blood pressure 195/86, heart rate 104, respiratory rate 19, oxygen saturation 96% on room air, and temperature 37.1°C. Laboratory values were significant for acute kidney injury (AKI) with new elevated creatinine (2.2), troponin-T elevation (0.57), creatine kinase (CK) total (3,124), and CK-muscle/brain (CKMB; 11.1). The patient was promptly started on insulin for hyperglycemia. An electrocardiogram was performed, showing regular rhythm with Q waves and ST elevation in V1 and V2 (Figure 1) without significant ST depressions, which was similar to an old electrocardiogram (Figure 2). As noted earlier, the patient had elevated troponins so interventional cardiology was promptly consulted through our transfer center. The interventional cardiology team reviewed all the electrocardiograms, and recommendations included non-emergent catheterization, to repeat the electrocardiogram in two hours, and to administer aspirin, intravenous (IV) heparin, IV nitroglycerin, and pain control.

**Figure 3:**
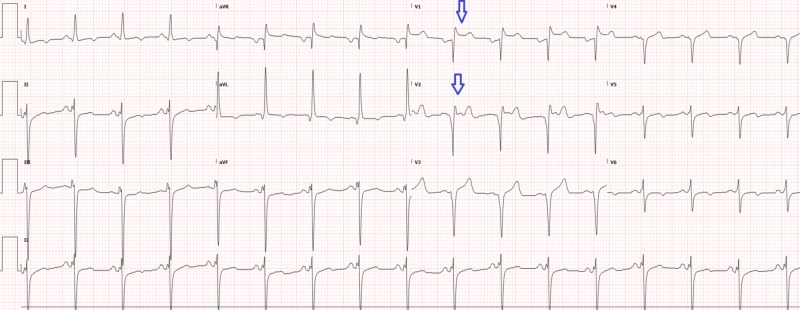
Electrocardiogram two hours after triage showing ST elevations (arrows)

The patient was observed in the emergency department for serial exams. Subsequently, the noninterventional cardiologist was consulted and he agreed with interventional cardiology’s assessment and plan for the case. A repeat two-hour electrocardiogram was similar to the initial electrocardiogram (Figure 3). During his course in the emergency department, the patient’s chest pain was resolving, but he started complaining of gradual onset abdominal and back pain that was refractory to all pain medications. He then started complaining of bilateral lower extremity paresis and anesthesia. On reevaluation, he had no palpable dorsalis pedis or posterior tibial pulse and no detectable pulse by Doppler or ultrasound. The compartments were soft and the distal extremities warm.

**Figure 4:**
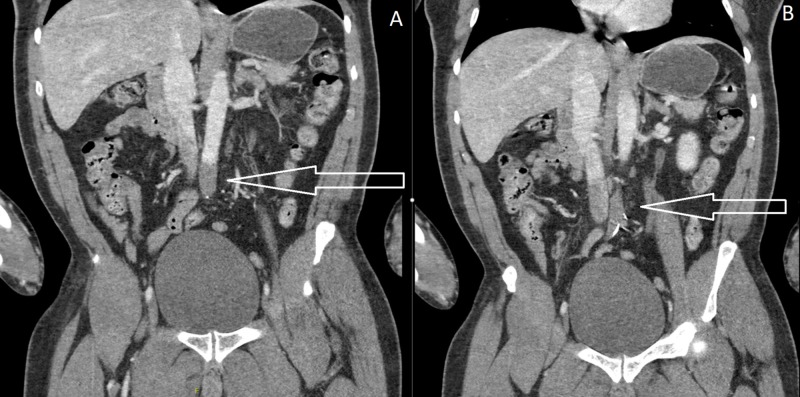
CT demonstrating distal aortic thrombus with filling defect (4A) and thrombus saddling into bilateral common iliac arteries (4B)

**Figure 5:**
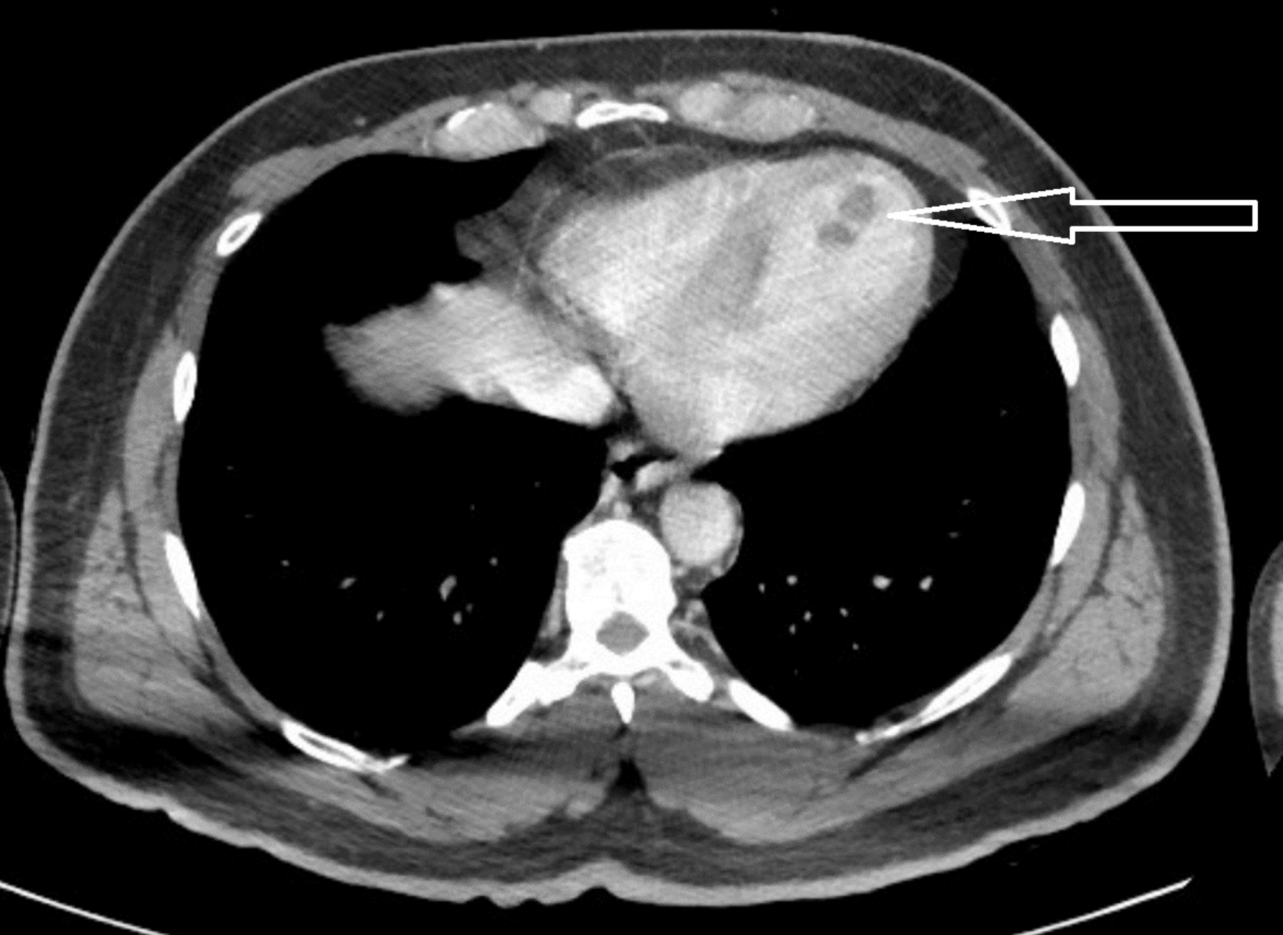
CT demonstrating intraventricular cardiac thrombus (arrow)

CT scans were performed, which showed a filling defect in the distal infrarenal aorta that was confirmed to be a distal infrarenal aortic saddle thrombus extending into the bilateral common iliac arteries (Figure 4). Additionally, an intraventricular thrombus was identified that was thought to be the cause of the aortic thrombus (Figure 5). Neurology was consulted at the time of intracardiac thrombus diagnosis and agreed to not give thrombolytics because the patient stated that he had a prior history of nonsurgical hemorrhagic cerebrovascular accident (CVA). However, they did agree with anticoagulation and to obtain an MRI/magnetic resonance angiography (MRA) brain/neck at admission to assess the showering of thrombi to the brain. The patient was transferred emergently to the operating room for vascular surgery to perform emergent thrombectomies and bilateral lower extremity four-compartment fasciotomies.

The patient had multiple tests during his hospital course, showing that the damage caused by the 1.7 cm cardiac intramural thrombus involvement had been diffuse. His MRI brain showed multiple subacute infarcts, indicating that the intramural cardiac thrombus had been present for weeks before he came to the emergency department. He also had right inferior pole renal infarcts that were age-indeterminate. On repeat testing, the size of the cardiac thrombus was shown to decrease after anticoagulation during hospital admission.

The patient’s hospital course included anticoagulation and hemodialysis due to rhabdomyolysis, causing acute tubular necrosis. He also had eventual stent placement during admission for subcritical CAD. The patient was in the intensive care unit for three weeks for frequent postoperative neurologic checks, respiratory support, and dialysis support; he was then discharged to inpatient rehabilitation six weeks after that. He now requires a wheelchair for mobility due to paraplegia and a urinary catheter for incontinence but does not require dialysis.

## Discussion

Acute aortic pathology, when seriously considered in the differential diagnosis by the emergency physician, must be ruled out with a contrast-enhanced CT. Most literature describing an acute saddle embolus of the aorta causing acute aortic occlusion is limited to older case reports and case series due to low incidence. The paucity of quality data on the condition is the hardest part of diagnosing the acute aorta for the emergency physician. Reducing the number of CT scans is a noble goal, but the loss of pulses and acute onset neurologic symptoms, such as in this case, present a clear indication to rule out an acute aorta.

Although cauda equina syndrome was briefly considered in this patient with acute onset low back pain and classic symptoms, it was replaced by acute aorta at the discovery of bilateral pulselessness. It was the bilateral absence of signals with a pencil Doppler that clinched the diagnosis of an acute aorta; the CT scan was just going to tell us what kind of acute aorta this patient would have.

A review of the literature shows case reports where cauda equina was considered in a patient with acute onset paraplegia and MRIs were ordered too, but the discovery of pulselessness led to the diagnosis of acute aortic thrombus [4-5]. Thus, Dopplers should be obtained in all patients with acute paraplegia, as palpation of pulses does not carry a high-enough level of certainty to rule out vascular conditions in cases of acute paraplegia [6-7].

Another consideration for vascular assessment is an ankle-brachial index of >0.9, which has diagnostic utility in cases of vascular compromise [8-9]. This case was clearly an acute vascular occlusion because the patient was pulseless and there were no collaterals present on CT scan [10]. Theoretically, in a similar case where a comprehensive vascular assessment was negative, the patient would have likely needed a stat MRI to rule out cauda equina or other spinal cord conditions.

Whether or not to obtain the CT scan with contrast rather than without contrast was an area of deliberation. The patient clearly had an acute kidney injury (AKI) with an increase of baseline creatinine from 1.3 to 2.2. One could balk at the thought of a contrast-enhanced CT scan with AKI, but when the differential consideration is so grave, the test must be done regardless of the patient’s creatinine. This decision to add contrast was another part of the workup where the true diagnosis of acute aortic bifurcation saddle thrombus could be missed because a non-contrast CT would likely not have shown the aortic occlusion; this is a case where diagnostic conviction is key to getting the patient treated. The ability of contrast media to cause acute kidney injury is a matter of debate in the literature so the benefits and risks must be weighed in each case.

One final point is that the patient was followed to CT scan and the emergency physician viewed the slices as they came on the screen while they were being produced by the CT scanner, as is often done during trauma alerts at most institutions. Attention was paid to the aorta in the scan, which was shown to be compromised. After calling the radiologist, the diagnosis was confirmed, and arrangements were made for the patient to be emergently transported to the operating room at another facility where vascular surgery would be waiting to do a thrombectomy. In a case where vascular compromise is considered, the physician should be in the CT room with the patient to minimize the time needed to arrange definitive treatment.

Overall, the patient did well for his diagnosis, especially considering that despite advances in vascular surgery and critical care over the past two decades, associated morbidity and mortality remain substantial with high rates of limb loss, acute renal failure, rhabdomyolysis, and death [11].

## Conclusions

Patients with chest pain are routine in the emergency department, but frequent reevaluation is a necessary, and often underperformed, action. Patients who start complaining of back pain and neurologic symptoms too require prompt and thorough neurovascular reevaluation because the possible differentials can be devastating. Doppler evaluation is essential in guiding the physician to an acute aorta over cauda equina and an eventual contrast-enhanced CT scan with stat vascular consultation and intervention; palpating pulses is simply not enough when the stakes are this high. The possibility of paraplegia, urinary retention, lifetime dependence on hemodialysis, or likely death are the major consequences of missing this diagnosis in the emergency department.
